# Craving among patients seeking treatment for substance use disorder

**DOI:** 10.1007/s44192-023-00049-y

**Published:** 2023-11-08

**Authors:** Mallory M. Cless, Natasia S. Courchesne-Krak, Kush V. Bhatt, Maria Luisa Mittal, Carla B. Marienfeld

**Affiliations:** Department of Psychiatry, University of California, San Diego, 8950 Villa La Jolla Drive, Suite C101, La Jolla, CA 92037 USA

**Keywords:** Cravings, Substance use, Future use, Clinical prognostication, Hispanic/Latinx, Adverse childhood experiences

## Abstract

**Background:**

Craving has been implicated as a central feature of addiction and a predictor of relapse. However, a complete understanding of how craving varies across patient populations is lacking. This study aimed to better inform the effective and accurate use of craving as a clinical prognostic tool for patients with substance use disorders (SUD).

**Methods:**

This cross-sectional study utilized information gathered on patients (n = 112) entering specialty treatment for a SUD. Craving in the prior 30 days was assessed with a single item with other intake questionnaires.

**Results:**

Patients who reported substance use in the last 30 days were more likely to report craving compared to patients who did not report substance use in the last 30 days (AOR = 6.86 [95% CI 2.17–21.7], p-value = 0.001). Patients who reported Hispanic/Latinx ethnicity were less likely to report craving compared to patients who did not report Hispanic/Latinx ethnicity (AOR = 0.28 [95% CI 0.08–0.95], p-value = 0.04). There was no association between craving and Adverse Childhood Events (OR = 1.03 [95% CI 0.84–1.25], p-value = 0.81).

**Conclusion:**

The association between recent substance use and craving supports previous findings. The observed variation in craving among patients who report Hispanic/Latinx ethnicity is novel and suggests socio-cultural influences and possibly genetic factors influencing reported craving amongst patients. Additional research is needed to further understand the underlying factors leading to this finding, in order for better utilization of craving as a clinical indicator across patient populations.

## Introduction

Craving is a well-documented experience amongst patients with substance use disorder (SUD), and a key clinical feature of the illness. A growing body of literature on the role craving plays in addiction resulted in changes made to the 2013 publication of the DSM-V. Craving is now included as part of the diagnostic criteria for SUD and is the only diagnostic feature also recognized as part of early and sustained remission [1]. The prominent role of craving throughout the course of addiction and recovery has led to research investigating its neurobiological basis, prognostic utility, and efficacy as a therapeutic target.

The prognostic power of craving has been disputed; however, several studies support craving as a predictor of two clinically significant outcomes: future use or “relapse” and treatment dropout [2–11]. At least two meta-analyses suggest that craving play a role in future use [12, 13] and the association between craving and future use holds true across different types of substances [14, 15]. Although craving appears to more strongly predict future use when assessed in close temporal proximity to use, it still appears to prognosticate relapse when patients are followed over longer periods of time [14, 16]. Several studies also demonstrate that craving serves as a barrier to treatment retention and predicts treatment dropout [3, 6, 8]. In order to improve treatment outcomes, it is critical to appropriately address craving in the clinical setting.

In order to sufficiently address craving and its clinical sequelae, it is imperative to identify factors associated with craving. Some potential factors have been studied previously. Negative affect is a well-documented predictor of craving [15, 17–19]. For example, one study in particular showed that stress induced craving compared to cue-induced craving was associated with shorter time to relapse [20]. Sociodemographic factors may similarly play a role in the presence of craving; however, the data at current is equivocal [23]. Some studies show increased craving in association with certain groups such as among women, while others have found no significant differences across these variables [23, 24].

In order to better inform effective and accurate use of craving as a clinical prognostic concept, this study examined several factors, including recent substance use, history of adverse childhood experience, and demographic factors in relation to reports of craving in the clinical setting. We hypothesized that craving would be associated with substance use within the past thirty days, a higher score on the Adverse Childhood Events (ACEs) questionnaire, and that craving would not be associated with sex, age, race, or ethnicity.

## Methods and design

### Data and study population

A cross sectional study was conducted utilizing data gathered from a convenience sample of patients entering outpatient treatment for SUDs at an addiction and mental health outpatient recovery and treatment program in Southern California. New prospective patients (ages 18 and older) were invited and consented to participate. Participants consented to the use of information collected during routine clinical care, such as demographics, self-report questionnaires, and structured evaluation questionnaires, for research purposes. Two-hundred patients were consented between November 1, 2017 and March 13, 2020, of which 112 were included in the study. Those not included were due to lack of response to some questionnaires. This study was approved by the Human Research Protection Program (HRPP) and the Institutional Review Board (IRB).

### Survey methods

Prior to their first visit, patients (n = 112) were assessed for clinical care with a series of self-report questionnaires, structured evaluation questionnaires, and semi-structured clinical interviews which included items inquiring about craving in the last 30 days and substance use in the last 30 days. Other information obtained included socio-demographic information (sex, age, race, Hispanic/Latinx ethnicity, marital status, education level, employment status, religious affiliation, self-reported substance use by substance type and current and past medical, psychiatric, and social history (medication history, family history, substance use history, system review, functioning, psychiatric symptoms). This information was gathered from hard-copy paper and pen surveys provided at the time of initial assessment and data extracted from electronic health records. To protect confidentiality, analytic databases created from the primary databases were stripped of personal identifiers and patients were assigned a unique study identification number.

### Measures

The primary outcome was reported craving in the past 30 days (yes/no). Patients were asked, “In the past 30 days, how much were you bothered by cravings or urges to drink alcohol or use drugs?” Patients were given the options of “not at all”, “slightly”, “moderately”, “considerably”, and “extremely.” This was then transformed into a dichotomous variable (yes/no). If patients answered “slightly” to “extremely” it was counted as a yes to craving in the past 30 days.

Sample covariates included sex (male/female), age (18–74), White race (yes/no), Hispanic/Latinx ethnicity (yes/no), married/significant other (yes/no), completed college (yes/no), employed full/part time (yes/no), religious (yes/no), ACEs score (0–10; high scores indicating more ACEs), driving under the influence charge (DUI; yes/no), history of arrest (yes/no), substance use in the last 30 days (yes/no), and self-reported problem by substance type (i.e., alcohol, opioids, stimulants, cannabinoids, sedatives). Questions on craving and recent substance use were pulled from the Brief Addiction Monitor (BAM) questionnaire, a 17- item self-report measure used to monitor substance use related behavior. The test–retest reliability of each item utilized from the BAM instrument in this study was previously shown to be acceptable. The “drug use in the past 30 days” item demonstrated good reliability when turned into a dichotomous variable, which was also done in this study [21]. Additionally, the specific items pulled from this questionnaire have shown to be valid when compared to other validated instruments [22]. These measures are commonly obtained during clinical intake in substance use treatment, and thus were reasoned to be relevant to clinicians interested in understanding craving and its associated factors.

### Analyses

Bivariate and multivariable logistic regression were used to determine factors associated with reported craving in the past 30 days. Continuous and categorical variables were summarized with means/standard deviation (SD) and totals/percentages respectively. Unadjusted and adjusted analyses of the covariate variables were conducted using Chi-square (X^2^) tests of significance. To determine the magnitude of the associations, unadjusted odds ratios (ORs) were calculated and reported. Covariates were assessed for significance using two-sided t-tests with 95% confidence intervals (CIs) and p-values. Covariates with confidence intervals that did not cross 1 and p-values ≤ 0.05 were considered significant and included in the final adjusted regression model. Multivariable logistic regression models were conducted to determine if variables found to be significant in the bivariate regression (i.e., patient reported Hispanic/Latinx ethnicity and substance use in the past 30 days) were associated with reported craving in the last 30 days. Standardized betas (β), standard errors (SE(β)), adjusted odds ratios (AOR), and the respective CIs and p-values were reported. All data analysis was conducted with SAS 9.4 (SAS Institute, Cary, North Carolina).

## Results

### Sample characteristics

Sample characteristics and the results of the bivariate analyses of factors associated with reporting craving in the last 30 days are shown in Table 1. Of the total 112 patients who consented to participate in this study and responded to the craving question, 92 (82.1%) reported craving in the last 30 days at their baseline initial outpatient appointment. Of the 112 participants, 53.6% were male, 75.9% reported White race, 17.3% reported Hispanic/Latinx ethnicity, and there was a mean age of 40.5 (standard deviation [SD] = 14.4, range 18–74 years of age). Single marital status was reported in 50.9%. The majority of patients completed college (52.7%), were employed (53.2%), and reported not being religious (64.5%). The mean ACE score was 2.7 (SD = 2.5; range 0–9). Most of the participants did not report a DUI (71.0%) or had not been arrested (64.5%). Thirty-day substance use (alcohol or other drugs) was reported in 81.8% and a self-reported alcohol problem was reported in 72.3%. Self-reported problems by substance type were highest for alcohol (72.3%), followed by opioids (33.9%), stimulants (29.5%), cannabinoids (20.5%), and sedatives (17.9%).

**Table 1:** Bivariate analysis of factors associated with reported craving among patients in a Southern California addiction clinic from November 1, 2017 to March 13, 2020 (n = 112)

Parameter	Total n (%)/mean (SD)	Reported cravings n (%)/Mean (SD)	No reported cravings n (%)/Mean (SD)	Odds ratio 95% (CI)	${X}^{2}$	P
All	112 (100.0)	92 (82.1)	20 (17.9)			
Sex						
Male	60 (53.6)	50 (54.4)	10 (50.0)	0.84 (0.32–2.21)	0.12	0.72
Female	52 (46.4)	42 (45.7)	10 (50.0)	__		
Age at intake (range 18–74)	40.5 (14.4)	40.0 (14.2)	43.0 (15.3)	0.99 (0.95–1.02)	0.71	0.40
White Race						
Yes	85 (75.9)	73 (79.4)	12 (60.0)	2.56 (0.92–7.16)	3.22	0.07
No	27 (24.1)	19 (20.7)	8 (40.0)	__		
Hispanic/Latino						
Yes	19 (17.3)	12 (13.3)	7 (35.0)	0.29 (0.10–0.86)	4.97	0.026
No	91 (82.7)	78 (86.7)	13 (65.0)	__		
Married/significant other*						
Yes	55 (49.1)	46 (50.0)	9 (45.0)	1.22 (0.46–3.23)	0.16	0.69
No	57 (50.9)	46 (50.0)	11 (55.0)	__		
Completed college						
Yes	59 (52.7)	50 (54.4)	9 (45.0)	1.46 (0.55–3.85)	0.57	0.45
No	53 (47.3)	42 (45.7)	11 (55.0)	__		
Employed full/part time						
Yes	58 (53.2)	49 (55.1)	9 (45.0)	0.67 (0.25–1.77)	0.66	0.42
No	51 (46.8)	40 (44.9)	11 (55.0)	__		
Religious						
Yes	38 (35.5)	30 (33.7)	8 (44.4)	0.64 (0.23–1.78)	0.75	0.39
No	69 (64.5)	59 (66.3)	10 (55.6)	__		
Adverse childhood experience score	2.7 (2.5)	2.70 (2.5)	2.55 (2.3)	1.03 (0.84–1.25)	0.06	0.81
DUI						
Yes	31 (29.0)	25 (28.7)	6 (30.0)	0.94 (0.33–2.72)	0.01	0.91
No	76 (71.0)	62 (71.3)	14 (70.0)	__		
Arrested						
Yes	38 (35.5)	32 (36.8)	6 (30.0)	1.36 (0.48–3.88)	0.32	0.57
No	69 (64.5)	55 (63.2)	14 (70.0)	__		
Thirty day use						
Yes	90 (81.8)	80 (87.9)	10 (52.6)	6.55 (2.18–19.6)	11.2	< 0.01
No	20 (18.2)	11 (12.1)	9 (47.4)	__		
Self-reported Alcohol Problem	81 (72.3)	69 (75.0)	12 (60.0)	2.0 (0.73–5.50)	1.80	0.18

^*^Married/significant other “yes” = married, living as married, significant other, in a relationship. *Married/significant other “no”* = single, separated, divorced, widowed. *SD* standard deviation, *CI* confidence interval, P-values based on Chi-squared (*X*^2^) tests of significance. Variable totals might not sum to column totals due to missing data

Of the participants who reported craving, 54.4% were male, 79.4% reported White race, 13.3% reported Hispanic/Latinx ethnicity, and there was a mean age of 40.0 (SD = 14.2). Single marital status was reported in 50.0%. The majority of patients completed college (54.4%), were employed (55.1%), and reported not being religious (33.7%). The mean ACE score was 2.7 (SD = 2.5; range 0–9). Most of the participants who reported craving did not have a DUI (71.0%) or had not been arrested (64.5%). Thirty-day use was reported in 81.8% and a self-reported alcohol problem was reported in 72.3%.

### Correlates of craving

In the bivariate analysis, reports of 30-day substance use (OR = 6.55, [95% CI 2.18–19.6], p-value =  < 0.001) and Hispanic/Latinx ethnicity (OR = 0.29, [95% CI 0.10–0.86], p-value = 0.026) were found to be significantly associated with reported craving. As such, sex, age, self-reported White race, marital status, college education, employment, religion, ACE score, history of DUI, arrest history, and self-reported alcohol problems were not associated with craving in this sample (Table 1). For all other self-reported substance use problems, there were cell counts < 5 and therefore significance was unable to be determined.

Multivariable logistic regression analysis of factors associated with reported craving in the last 30-days are shown in Table 2. Patients who reported Hispanic/Latinx ethnicity were less likely to report craving compared to patients who did not report Hispanic/Latinx ethnicity (AOR = 0.28 [95% CI 0.08–0.95], p-value = 0.04). Patients who reported substance use in the last 30 days were more likely to report craving compared to patients who did not report substance use in the last 30 days (AOR = 6.86 [95% CI 2.17–21.7], p-value = 0.001). There was no association between craving and Adverse Childhood Events (OR = 1.03 [95% CI 0.84–1.25], p-value = 0.81).

**Table 2:** Multivariable logistic regression analysis of factors associated with reported craving among patients in a Southern California addiction clinic from November 1, 2017 to March 13, 2020 (n = 108)

Parameter	*B*	SE (β)	${X}^{2}$	Adjusted Odds Ratio (95% CI)	*P*
Hispanic/Latino	− 1.26	0.62	4.20	0.28 (0.08–0.95)	0.04
30-day substance use (ref = no)	1.93	0.59	10.7	6.86 (2.17–21.7)	0.001

*B* unstandardized beta*,* *β* standardized beta, *SE* standard error, *CI* confidence interval. P-values based on Chi-squared (*X*^2^) tests of significance

## Discussion

In this study we evaluated factors associated with craving among those with an SUD in an outpatient treatment setting. We found that patients who reported active substance use in the past 30 days were more likely to report craving in the past 30 days. We also found that those who reported Hispanic/Latinx ethnicity were less likely to report craving in the past 30 days. Sex, age, patient reported White race, marital status, completing college, employment, religion, ACE score, history of DUI, arrest history, and self-reported alcohol problem were not associated with craving in this sample.

The goal was to evaluate various clinically relevant factors that may be associated with craving to better understand the significance of reported craving in a clinical setting. This study contributes further knowledge suggesting that craving is an important clinical marker of future use, and offers new information regarding variations in craving across ethnic groups, a topic which remains incompletely understood.

A large number of studies evaluate craving and future substance use, but few evaluate the relationship between craving and substance use in the 30 days preceding treatment entry. We found that those who reported any substance use in the last 30 days were significantly more likely to report craving during that same time. This finding supports our hypothesis and aligns with prior research showing craving to be positively correlated with the number of heavy drinking days, severity of alcohol dependence, and overall substance use [23]. Craving reported on treatment entry thus indicates a need for evaluation of withdrawal symptoms and recent use indicates a need for craving assessment.

The observed findings show that patients who report Hispanic/Latinx ethnicity and have a SUD were overall less likely to report craving, even when accounting for recent substance use. This finding may be explained by psychosocial factors influencing report measures, and possibly genetic factors, or both. Racial/ethnic disparities regarding substance use are well documented, particularly among Hispanic/Latinx individuals [24, 25]. Prior research demonstrates that Hispanic/Latinx patients underutilize SUD treatment due to stigma [25]. Latinx individuals experience higher levels of negative bias, worse social consequences, and decreased social support regarding substance use compared to patients who report White race [25–28]. Furthermore, the perception of low treatment efficacy, in part influenced by concerns that providers lack ‘real world’ experience, may play a role in comfort level with reporting and disclosing craving [27].

Genetic factors may also play a role in the lower reported cravings amongst Hispanic/Latinx patients. In patients with alcohol use disorder, variation in efficacy of naltrexone for mitigating craving among individuals of White race or African American ethnicity is thought to be attributable to a specific polymorphism [22]. This suggests a possible genetic basis for difference in the experience of craving amongst varying populations [22]. Polymorphisms found in other genes, such as those in the dopamine reward pathway and those encoding for protein alpha-synclein, are associated with increased craving amongst those with SUD [29–31]. These genetic variations are suspected to be specific to craving and distinct from other genetic pathways underlying the SUD phenotype [30]. With that in mind, one study found that there was no difference in alcohol craving between patients in Mexico, Argentina, Poland, and the USA [32]. As research continues to investigate the genetic basis of craving and addiction, we recommend further evaluation across race and ethnicity. Elucidating genetic markers associated with lower levels of craving can help in the development of neurobiological treatment targets in the future.

This study is the first known to evaluate the relationship between ACE score and craving. Past studies have found emotional reactivity/dysregulation and negative affect, such as anxiety and stress, to be correlated with craving and SUD [2, 5, 33]. Excessive stress signaling is thought to prime the brain’s reward system for addictive behaviors [33]. Given the role of the reward system in mediating craving and the known negative effects of adverse childhood experiences on emotional regulation, we suspected ACE scores would be associated with increased reports of craving. However, no significant relationship was found, contrary to what was hypothesized. There are several possible reasons why this finding may not have been observed. It may indeed be that ACEs are not associated with reported craving, and that the neurobiological and psychological pathways do not align in a way which leads to a direct correlation. Further studies should explore this relationship in order to assess if this finding is replicated. Conversely, certain population that were not represented in our study may demonstrate correlations between ACEs and craving. Our study population included those age 18 and above, but individuals below that age were not included and remain to have unknown clinical correlates of craving. This would be an interesting area of future research given the rapidly developing cortical brain networks at these younger ages amidst more recent or even ongoing exposure to childhood traumatic experiences. To more comprehensively understand the relationship between ACEs and craving, it may be necessary to examine subpopulations beyond what was explored in this study.

There are several limitations to consider when interpreting the results of this study. Craving is a complex phenomenon that is not always easily and accurately assessed, and thus, the validity of craving measurements has been disputed [11]. We utilized a single item question to assess craving and transformed answers into a dichotomous outcome of yes or no. This could have impacted the reliability of the craving measurement and restricted the maximum possible correlation. This study was also limited to those seeking specialty outpatient SUD treatment, and therefore, may not be generalizable to the experience of craving across all those with a SUD, given many never seek treatment or have access to treatment. Lastly, the sample size was not large enough to evaluate the individual effects of substance type (stimulants, opioids, etc.) on craving. We highlighted several areas of research needed to expand on the results found here, including the genetic underpinnings of race and craving, sociocultural factors influencing reports of craving, and the impacts of ACE on craving. These findings may influence how we approach craving as a treatment target.

## Conclusion

In summary, craving is a central feature of addiction and has been implicated as a predictor of future use. This study attempted to elucidate clinical correlates of craving in order to further our understanding of it as a clinical prognostic tool. We found that patients who report Hispanic/Latinx ethnicity were less likely to report craving. We also found that individuals who had used within the last 30 days were more likely to report craving. Our results may help clinicians when using reported craving as a tool for treating various populations with SUDs. Further studies are warranted to explore this topic and continue to deepen our understanding of craving as a clinically useful tool.

## Data Availability

The data analyzed during the current study are available from the corresponding author on reasonable request.
